# Alginate–Aluminosilicate Clay Beads for Sustained Release of Chlortetracycline Hydrochloride: Development and In Vitro Studies

**DOI:** 10.3390/gels11110921

**Published:** 2025-11-18

**Authors:** Aicha Nour Laouameria, Meriem Fizir, Sami Touil, Amina Richa, Nassima Benamara, Houda Douba, Liu Wei, Djamila Aouameur, Houria Rezala, Attila Csík, Tamás Fodor

**Affiliations:** 1 Doctoral School of Chemistry, University of Debrecen, Egyetem tér 1, H-4032 Debrecen, Hungary; 2 HUN-REN Institute for Nuclear Research, Bem tér 18/c, 4026 Debrecen, Hungary; csik.attila@atomki.hu (A.C.); fodor.tamas@atomki.hu (T.F.); 3 Laboratory of Precision Agriculture, Environment and Sustainable Development, Khemis Miliana University, Khemis Miliana 44225, Algeria; s.touil@univ-db.dz (S.T.); a.richa@univ-dbkm.dz (A.R.); nassimabenamara801@gmail.com (N.B.); d.aouameur@univ-dbkm.dz (D.A.); 4 Laboratoire de Valorisation des Substances Naturelles, Khemis Miliana University, Khemis Miliana 44225, Algeria; h.douba@univ-dbkm.dz (H.D.); h.rezala@univ-dbkm.dz (H.R.); 5 Laboratory of Physical Chemistry of Material Interfaces Applied to the Environment, University of Saad Dahlab Blida 1, Blida 09000, Algeria; 6 School of Pharmaceutical Business, Zhejiang Pharmaceutical University, Ningbo 315500, China; liuw@zjpc.net.cn

**Keywords:** alginate beads, halloysite nanotubes, kaolin, drug delivery, chlortetracycline hydrochloride, sustained release, adsorption capacity

## Abstract

This study reports the preparation of alginate (Alg) beads incorporating different amounts of halloysite nanotubes (HNTs) and kaolin (K) in the presence of Ca^2+^ ions to compare their drug loading and release behaviors. The resulting composites, HNTs@Alg and K@Alg, were characterized using FTIR, SEM–EDS, XRD, and XPS analyses. Chlortetracycline hydrochloride (CTC) was employed as a model antibiotic to evaluate their drug delivery performance. The concentration of Alg and the incorporation of HNTs or K markedly influenced the adsorption capacity and release profile. The maximum drug loading capacities were 48.12 ± 1.4 mg/g for HNTs, 40.1 ± 1.2 mg/g for K, 59.85 ± 2.3 mg/g for HNTs@Alg-1 (1 g HNTs and 1% Alg), and 68.74 ± 2.1 mg/g for K@Alg-1 (1 g K and 1% Alg). The inclusion of Alg enhanced sustained release, extending beyond 100 h. Among the composite beads, HNTs@Alg-1 showed superior CTC release behavior compared to K@Alg-1. Furthermore, antibacterial assays confirmed that the CTC-loaded beads effectively inhibited *E. coli* and *S. aureus*, demonstrating maintained drug activity after encapsulation. Both systems effectively prolonged CTC release and exhibited antibacterial efficacy, highlighting their potential as controlled drug delivery matrices for wound treatment applications.

## 1. Introduction

Drug delivery is the process by which drug molecules are transported to their target sites while minimizing immunogenicity and biological inactivation. The development of new drug delivery systems (DDS) is crucial due to the increasing demand for efficient therapeutic delivery [1]. DDS has gained significant attention in biomedical research for its potential to reduce side effects, minimize dosing frequency, and prevent gastrointestinal intolerance [2]. Among various antibiotics, tetracycline hydrochloride (TC) is widely used against infections caused by species such as Chlamydia, Mycoplasma, Rickettsia, and other Gram-positive and Gram-negative bacteria [3]. Chlortetracycline hydrochloride (CTC), a common antibiotic from the tetracycline class, is used to treat various skin conditions, including acne and bacterial infections [4].

Polysaccharide-based hydrogels, such as those derived from alginate or chitosan, have been extensively studied for drug delivery applications due to their low cost, biocompatibility, and eco-friendly nature. Alginate, a naturally occurring anionic polymer derived from brown seaweed, is frequently used in hydrogel formation for drug delivery. Alginate hydrogels can be cross-linked in various ways, and their structural similarity to extracellular matrices makes them suitable for wound healing, bioactive agent delivery, and cell transplantation [5]. However, nanocomposite hydrogels often suffer from drawbacks such as instability, poor mechanical properties, and rapid drug release [3].

Clay minerals have gained importance in drug delivery applications due to their unique structural and compositional properties. Halloysite nanotubes (HNTs) and kaolin (K), two hydrated phyllosilicates, commonly found in soils and sedimentary rocks, are among the most studied clay materials for drug delivery systems (DDS) because of their biocompatibility, stability, and ability to encapsulate drug molecules [6,7]. HNTs, naturally occurring aluminosilicate clays, possess an external siloxane (Si–O–Si) surface and internal hydroxyl (Al–OH) groups [8,9]. Both kaolin and halloysite belong to the phyllosilicate group and share a layered structure; however, halloysite’s additional water molecules affect its basal spacing and morphology, providing efficient sites for drug loading and controlled release [10].

While montmorillonite (MMT) is widely used as a pharmaceutical excipient, kaolin (K) and halloysite nanotubes (HNTs) frequently offer superior performance in advanced drug delivery systems due to their enhanced biocompatibility, lower cost, and more controllable physicochemical properties. A major limitation of MMT is its high cation exchange capacity (CEC) combined with substantial swelling in aqueous environments. These characteristics can trigger an excessively rapid initial burst release of loaded drug molecules and can compromise the stability of formulations by altering viscosity and particle dispersion over time. In contrast, kaolin possesses a markedly lower CEC and exhibits a non-swelling nature, which together contribute to a more predictable and stable drug release profile. These features make kaolin particularly advantageous for designing durable, long-term release matrices with reduced risk of premature drug leakage [11]. Halloysite nanotubes provide an additional structural advantage arising from their naturally occurring tubular morphology. The hollow lumen enables high drug-loading efficiency, while the aluminosilicate shell functions as a diffusional barrier that inherently supports sustained release, an attribute not achievable with the plate-like structure of MMT. Moreover, the differential surface chemistry of HNTs, characterized by an alumina-rich inner surface and a silica-rich outer surface, allows for selective and independent functionalization. This tunability permits fine control over drug-carrier interactions and release kinetics in ways that conventional layered clays cannot offer [12]. Collectively, these attributes position kaolin and halloysite as compelling, and often superior alternatives to montmorillonite for drug-delivery applications requiring precise modulation of release behavior, enhanced formulation stability, and specialized encapsulation capabilities [13].

Inspired by previous research, this study focuses on developing alginate bead composites incorporating Algerian halloysite nanotubes and kaolin, referred to as HNTs@Alg and K@Alg. Using chlortetracycline hydrochloride as a model drug, this work investigates the drug uptake mechanisms, sustained release behaviors, and antibacterial activity of these composites. Characterization techniques, including X-ray diffraction (XRD), Fourier-transform infrared spectroscopy (FTIR), scanning electron microscopy (SEM), energy-dispersive X-ray spectroscopy (EDS), and X-ray photoelectron spectroscopy (XPS), were employed to analyze the synthesized materials and elucidate the adsorption mechanism. The effects of key parameters such as contact time, adsorbent dosage, pH, initial CTC concentration, and temperature on the loading capacity of beads were also optimized. This comparative study provides insights into how the structural differences between HNTs and K influence their performance in alginate-based drug delivery systems.

## 2. Results and Discussion

This section presents the characterization and performance evaluation of the prepared alginate-based composite beads incorporating halloysite nanotubes and kaolin. The formulations were synthesized by ionic crosslinking of sodium alginate (SA) with Ca^2+^ ions, producing Alg beads, HNTs@Alg beads, and K@Alg beads with varying Alg and clay ratios. Chlortetracycline hydrochloride was used as a model drug to assess their adsorption, loading, and release behaviors. The materials were subsequently characterized using FTIR, XRD, SEM–EDS, and XPS analyses to confirm the successful incorporation of the clays into the alginate matrix and to verify drug uptake within the composite beads. In addition, the antibacterial performance of the CTC-loaded beads was evaluated to determine the bioactivity of the released drug.

### 2.1. Characterization

#### 2.1.1. FTIR Analysis

**Figure 1:**
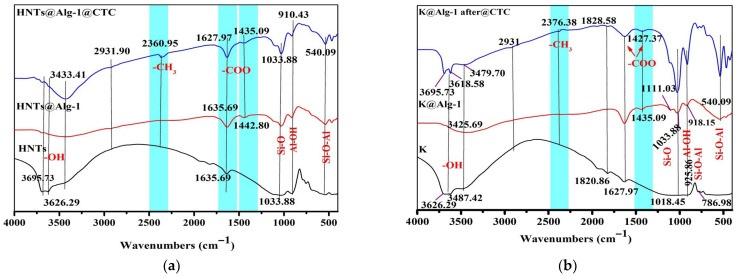
FT-IR Spectra of: (**a**) HNTs, HNTs@Alg-1, and post-adsorption HNTs@Alg-1; (**b**) K, K@Alg-1, and post-adsorption K@Alg-1.

The infrared spectra of HNTs, HNTs@Alg-1 before and after CTC adsorption are presented in Figure 1a. In the FTIR spectrum of HNTs, characteristic bands at 3695 cm^−1^ and 3626 cm^−1^ correspond to the stretching vibrations of Al–OH groups. The Si–O stretching mode appears at 1033 cm^−1^, while the bending vibration of Al–OH in HNTs is observed at 910 cm^−1^. Additionally, the signals at 540 cm^−1^ and 455 cm^−1^ correspond to the bending vibrations of Al–O–Si and Si–O–Si, respectively, whereas the peak at 424 cm^−1^ is attributed to the Si–O deformation mode. The FTIR spectrum of HNTs@Alg-1 composite beads exhibited strong absorption bands at 1635 cm^−1^ and 1442 cm^−1^, which are assigned to the asymmetric and symmetric stretching vibrations of the –COO functional groups of alginate, respectively. A broad absorption band at 3433 cm^−1^ is associated with the –OH groups of sodium alginate and adsorbed water. Sodium alginate typically exhibits an absorption band at 1029 cm^−1^, corresponding to the C–O–C (cyclic ether) stretching vibration. However, due to the close proximity of the Si–O stretching band in HNTs (1033 cm^−1^), these signals overlap, resulting in a broad band at 1033 cm^−1^ in the HNTs@Alg-1 spectrum. The incorporation of HNTs into alginate did not induce significant shifts in peak positions or the emergence of new peaks. The FTIR spectra of HNTs@Alg-1 bio-nanocomposite beads contained all characteristic absorption bands of both halloysite nanotubes and alginate, suggesting no chemical interaction between the two components. Consequently, the active sites of HNTs and the mannuronic acid residues of alginate (particularly those not involved in the gelation process with Ca^2+^) likely remain available for further applications [14].

The infrared spectra of K, K@Alg-1, before and after CTC adsorption, are presented in Figure 1b. The FTIR spectrum of K exhibits a characteristic Si–O stretching vibration at 1018 cm^−1^. The peak at 786 cm^−1^ corresponds to the stretching vibration of Si–O–Al, while the bands at 925 cm^−1^ and 3695 cm^−1^ are attributed to Al–OH stretching vibrations from the alumina sheets of K. Sodium alginate functional groups are evident in the spectrum, with peaks at 1435 cm^−1^ and 1627 cm^−1^, corresponding to the symmetric and asymmetric stretching vibrations of carboxylate anions (–COO^−^), respectively. The –OH stretching bands appear at 3677 cm^−1^ and 3487 cm^−1^, which can be attributed to the interaction between hydroxyl groups and the Al sites in K clay [15]. In the FTIR spectrum of K@Alg-1, a broad absorption band at 3425 cm^−1^ is assigned to the –OH groups of sodium alginate and absorbed water. The characteristic FTIR bands of both sodium alginate and K were retained, confirming the successful formation of the K@Alg-1 composite beads [16]. Following CTC adsorption, the FTIR spectra of HNTs@Alg-1 and K@Alg-1 composite beads retained most of the characteristic bands of the composite material, with noticeable shifts in wavenumber, suggesting the interaction between the adsorbent and CTC molecules. Additionally, a distinct band at 2931 cm^−1^, assigned to the CH_3_ stretching vibration in aromatic rings (see SI, Figure S1) was observed, confirming the presence of CTC within the composite structure [17].

#### 2.1.2. XRD Analysis

**Figure 2:**
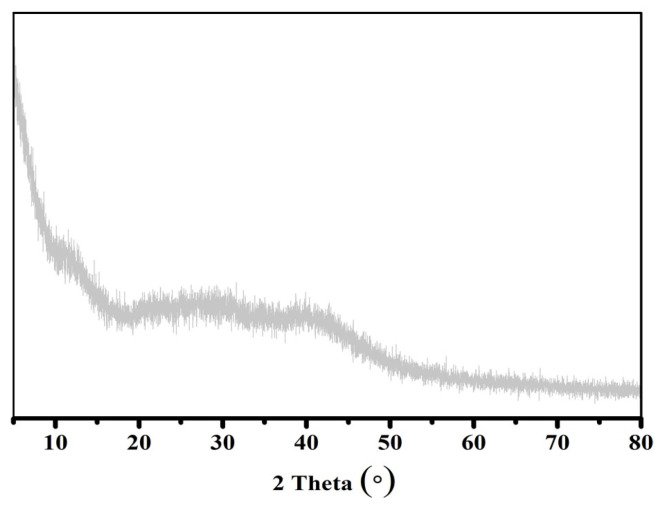
XRD Patterns of Alg beads.

**Figure 3:**
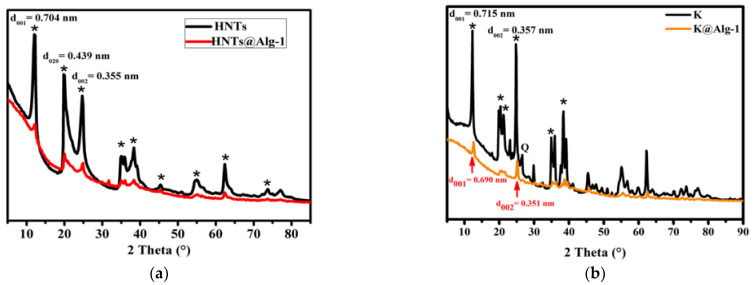
XRD Patterns of: (**a**) HNTs and HNTs@Alg-1, where (*) indicates HNTs; (**b**) K and K@Alg-1, where (*) indicates K.

The XRD patterns provided important insights into the structural characteristics of the alginate beads (Figure 2) and the incorporation of HNTs and K within the alginate gel matrix (Figure 3). The XRD profile of the pure alginate beads exhibited an amorphous pattern, indicating the absence of crystalline domains [18]. Analysis of the HNTs samples (Figure 3a) revealed a distinct diffraction peak at 2θ = 12.55°, corresponding to a basal spacing of 0.70 nm, as calculated using Bragg’s law. This confirms the presence of halloysite nanotubes in their expected hexagonal Al_2_Si_2_O_5_(OH)_4_ structure, without interlayer water [14,19]. Additional characteristic HNTs peaks were observed at 20.20°, 25.04°, 35.25°, 38.54°, 55.12°, and 62.43° in both the HNTs and HNTs@Alg-1 samples, indicating that the native structure of the HNTs was retained even after incorporation into the alginate matrix. In contrast, the XRD pattern of K displayed a slightly larger basal spacing of approximately 0.72 nm (7.2 Å), compared to the 0.70 nm (7 Å) spacing observed for halloysite [20]. Distinct K peaks appeared at 12.36°, 20.43°, 21.42°, 24.86°, 35.90°, and 38.46°, with the most intense peak at 24.86°. A diffraction peak at 2θ = 26.6° further confirmed the presence of quartz as a minor impurity within the K sample (Figure 3b) [21]. Notably, although the intensities of these characteristic peaks were reduced, they remained visible in the XRD patterns of the final alginate gel beads. This indicates that both HNTs and K were successfully and uniformly dispersed within the alginate matrix during the blending process. These findings are consistent with previous studies on alginate–clay composites [22], further validating the effective incorporation of HNTs and K into the gel bead structure.

#### 2.1.3. SEM Analysis

**Figure 4:**
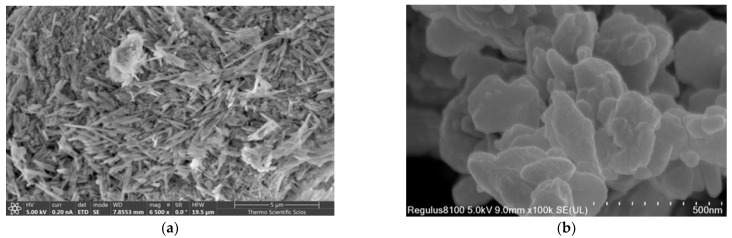
SEM images of: (**a**) HNTs; (**b**) K.

Scanning Electron Microscopy was employed to investigate the morphological characteristics of raw HNTs and K, as shown in Figure 4. The SEM images clearly reveal the rolled nanotubular structure of halloysite (Figure 4a), with lengths of 0.5–2 μm. Furthermore, the tubular particles appeared straight, with open-ended lumens, consistent with observations reported in previous studies [23]. In Figure 4b, SEM analysis of K reveals irregularly shaped crystalline structures with uneven edges, and an approximate particle size of 0.2 μm.

**Figure 5:**
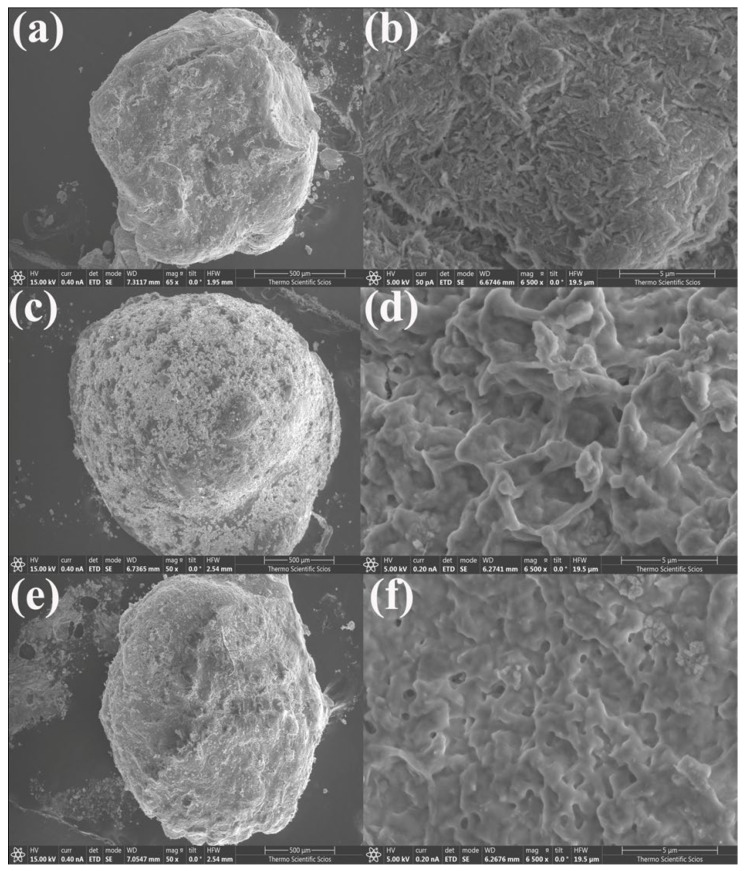
SEM images of: (**a**,**b**) HNTs@Alg-1; (**c**,**d**) HNTs@Alg-2; and (**e**,**f**) HNTs@Alg-3.

**Figure 6:**
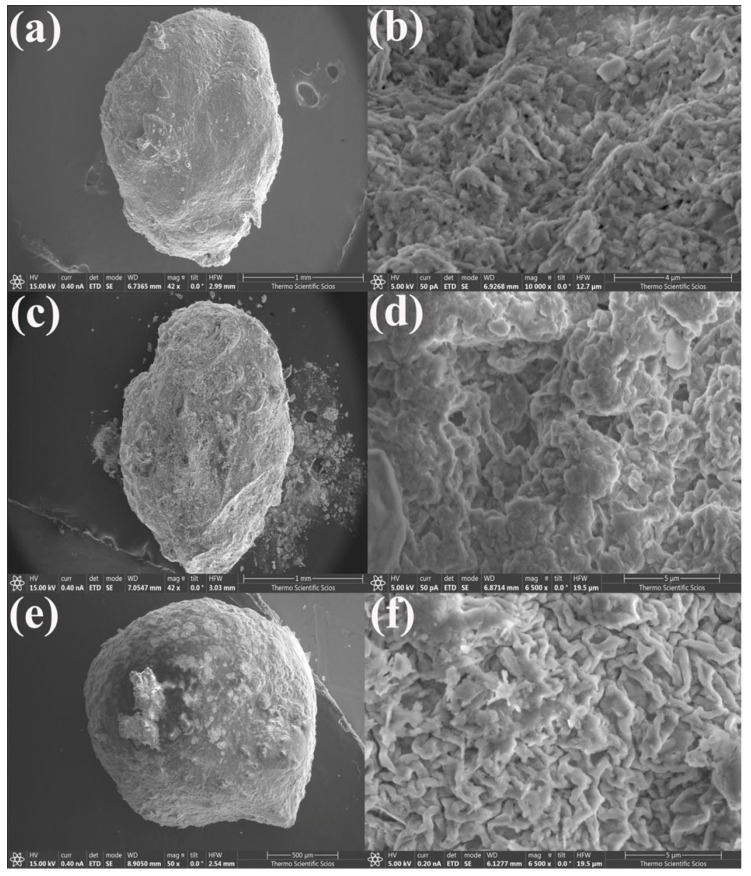
SEM Images of (**a**,**b**) K@Alg-1; (**c**,**d**) K@Alg-2; and (**e**,**f**) K@Alg-3.

The surface morphology of the synthesized K@Alg and HNTs@Alg composite beads was analyzed using SEM, as shown in Figure 5 and Figure 6. The images indicate that the beads exhibit an irregular surface texture, characterized by cracks, undulations, folds, and pores. Additionally, the non-uniform microstructure of the beads consists of polyhedral particles of varying sizes, aligning with findings from previous research [24,25]. The SEM analysis of the composite beads revealed a noticeable variation in surface morphology, particularly in the porosity of the structures, which appears to be strongly influenced by the alginate concentration. As observed in the SEM images, beads with a lower alginate concentration exhibited a higher density of pores (Figure 5b,d and Figure 6b,d), whereas those with an increased alginate content demonstrated a more compact structure with fewer visible pores (Figure 5f and Figure 6f).

This phenomenon can be attributed to the gelation mechanism of alginate in the presence of Ca^2+^ ions. At lower alginate concentrations, the polymer network forms a loose and open structure, allowing for the creation of larger and more numerous pores. However, as the alginate concentration increases, the polymer chains become more densely packed, leading to a tighter network with reduced pore formation. This densification effect results from a higher availability of alginate functional groups, which enhances crosslinking interactions with Ca^2+^, thereby restricting the formation of large pores. Additionally, the reduced porosity at higher alginate concentrations may also affect the diffusion and adsorption properties of the beads. A denser structure could limit mass transfer, potentially influencing the adsorption capacity of the composite beads. This structural evolution aligns with previous studies, which report that an increase in alginate concentration leads to the formation of more compact and mechanically stable hydrogel networks [26]. These findings highlight the importance of optimizing alginate content for controlled porosity and functional performance in adsorption and drug delivery applications.

**Figure 7:**
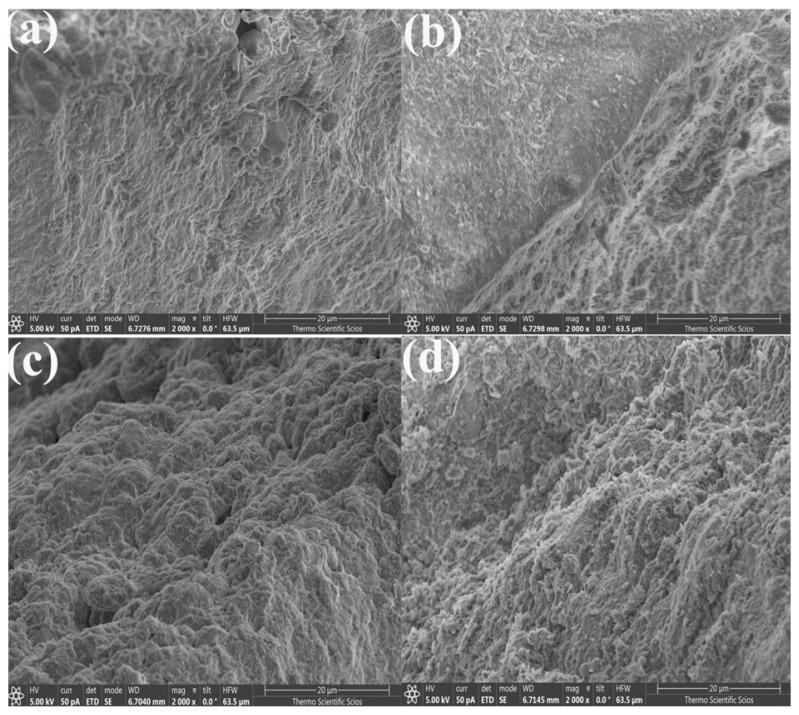
SEM Images of: (**a**) HNTs@Alg-1; (**b**) post-adsorption HNTs@Alg-1; (**c**) K@Alg-1; and (**d**) post-adsorption K@Alg-1.

After CTC adsorption, SEM analysis of the composite beads (Figure 7) revealed that the pleated voids on the bead surfaces were filled with a substantial number of fine particles. These particles are likely formed due to the flocculation of CTC molecules, resulting from local supersaturation on the bead surface. Similar morphological transformations following adsorption have been reported in previous studies [15].

#### 2.1.4. EDS Analysis

**Figure 8:**
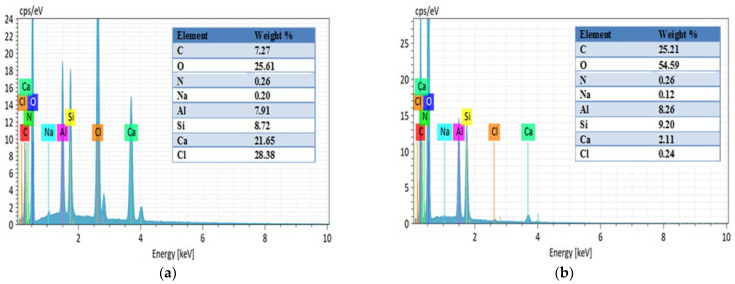
EDS analysis of: (**a**) HNTs@Alg-1; (**b**) post-adsorption HNTs@Alg-1.

**Figure 9:**
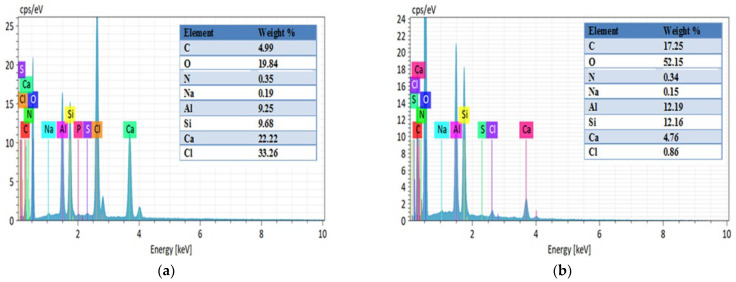
EDS analysis of: (**a**) K@Alg-1; (**b**) post-adsorption K@Alg-1.

The EDS analysis presented in Figure 8 and Figure 9 reveals significant compositional changes before and after CTC adsorption, highlighting key aspects of the adsorption mechanism. Following adsorption, the carbon (C) content in HNTs@Alg-1 increased from 7.27% to 25.21%, and oxygen (O) from 25.61% to 54.59%. Similarly, in K@Alg-1, carbon (C) increased from 4.99% to 17.25%, and oxygen (O) from 19.84% to 52.15%. These increases confirm the successful loading of CTC, which contains carbon-based structures and oxygen-rich functional groups.

A notable decrease in calcium (Ca) content was also observed: from 21.65% to 2.11% in HNTs@Alg-1 and from 22.22% to 4.76% in K@Alg-1. This reduction may be attributed to interactions between calcium ions and CTC during the adsorption process, as well as to the apparent dilution of elemental content due to the presence of the adsorbed CTC layer.

#### 2.1.5. XPS Analysis

**Figure 10:**
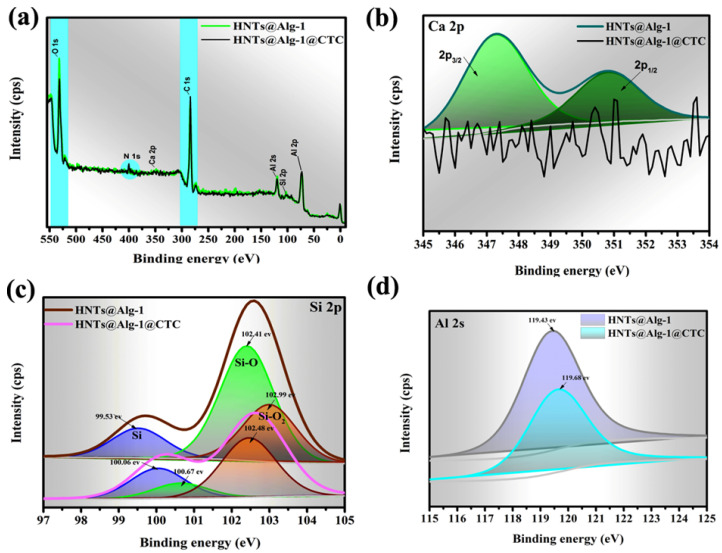
(**a**) Wide-scan XPS spectra of HNTs@Alg-1; (**b**) Ca 2p spectra; (**c**) Si 2p spectra; and (**d**) Al 2s spectra before and after CTC loading.

**Figure 11:**
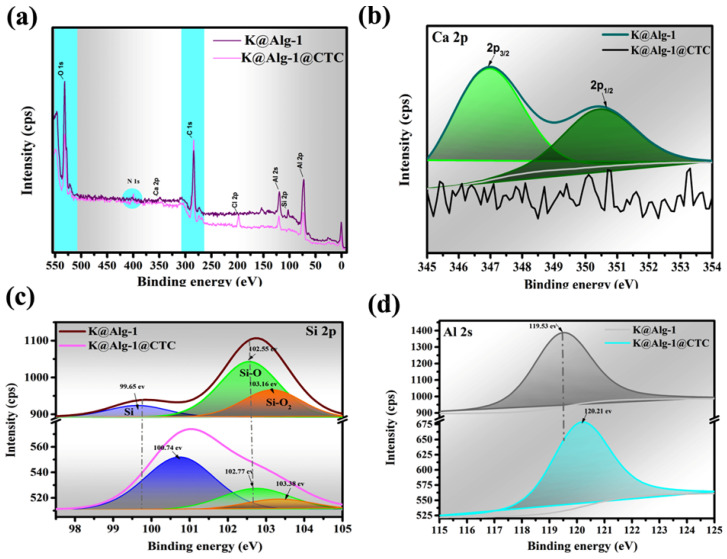
(**a**) Wide-scan XPS spectra of K@Alg-1; (**b**) Ca 2p spectra; (**c**) Si 2p spectra; and (**d**) Al 2s spectra before and after CTC loading.

The high-resolution XPS spectra of K@Alg-1 and HNTs@Alg-1 beads before and after CTC adsorption revealed that the primary elements in both composites were carbon (C), oxygen (O), aluminum (Al), silicon (Si), and calcium (Ca) (Figure 10 and Figure 11). Following CTC adsorption, a distinct peak emerged near 400 electron volt (eV), corresponding to the N 1s signal, confirming the successful immobilization of CTC onto the composite surfaces. Additionally, an increase in the intensity of the C peak further supported the adsorption process [27]. In contrast, the O peak intensity decreased, likely due to its participation in electrostatic interactions and hydrogen bonding with CTC molecules.

To elucidate the adsorption mechanism, the variations in Ca, Al, and Si signals before and after CTC adsorption were analyzed. Notably, the Ca 2p peak disappeared post-adsorption in both composites (Figure 10b and Figure 11b), suggesting that hydroxyl (-OH) and ketone (C=O) groups in CTC chemically interacted with Ca^2+^ ions, potentially forming a complex or precipitate [28]. This interaction could alter the electronic environment of calcium, leading to its peak disappearance. Additionally, CTC’s hydroxyl, carbonyl, and amino groups likely donated electrons to Ca^2+^, facilitating metal complex formation through cation-π and cation-n interactions [17], a trend also observed in metal–organic framework MIL-100 containing iron (MIL-100(Fe))@chitosan composite beads during CTC adsorption [28].

The Si 2p peaks at 99.65, 102.55, and 102.99 eV for HNTs@Alg-1 (Figure 10c) and 99.65, 102.41, and 103.16 eV for K@Alg-1 (Figure 11c), attributed to Si, Si-O, and Si-O_2_ groups [29], shifted to 100.06, 100.67, 102.48 eV, and 100.74, 102.77, 103.38 eV, respectively. This shift indicates the involvement of Si-containing groups in CTC adsorption via electrostatic interactions between Si^4+^ and CTC^−^, hydrogen bonding between Si-O/Si-O_2_ and hydroxyl groups in CTC, and complexation interactions. Furthermore, the Al 2p peak shifted by approximately 0.25 eV and 0.68 eV for HNTs@Alg-1 (Figure 10d) and K@Alg-1 (Figure 11d), respectively, suggesting that Al also contributed to CTC adsorption through complex formation [30].

### 2.2. Effect of Experimental Parameters on Loading Capacity of CTC

**Figure 12:**
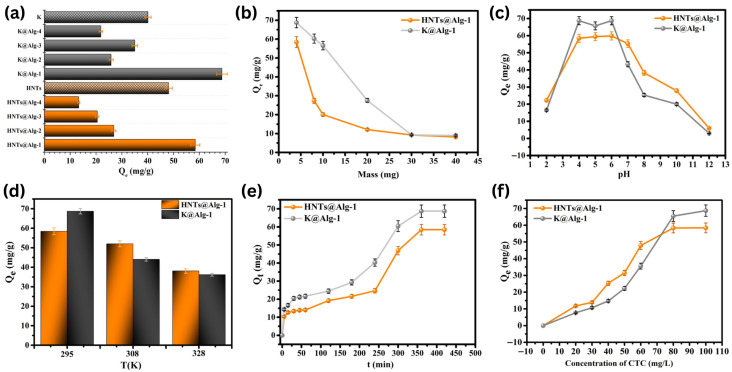
(**a**) Loading capacity of different alginate bead series; (**b**) Effect of bead mass; (**c**) Effect of solution pH; (**d**) Effect of temperature; (**e**) Effect of contact time; and (**f**) Effect of CTC concentration on the loading capacity of CTC on HNTs@Alg-1 and K@Alg-1 beads. Error bars represent standard deviations (SD) of replicate measurements.

The loading capacity of CTC onto the studied clays and beads is presented in Figure 12a. HNTs, K, and their corresponding alginate-based beads prepared with 1% alginate solution and 1 g of HNTs or K exhibited the highest adsorption capacities, reaching 58.43 ± 1.7 mg/g and 68.74 ± 2.1 mg/g for HNTs and K composites, respectively. However, the adsorption capacity of HNTs@Alg-2, HNTs@Alg-3, and HNTs@Alg-4 beads was lower than that of raw HNTs, likely due to intraparticle diffusion limitations caused by the denser bead matrix, which restricted CTC diffusion at the early adsorption stage [31]. A similar trend was observed in K-based nanocomposites. Moreover, HNTs demonstrated a higher adsorption capacity than K, attributed to their unique structure, which facilitates the adsorption of organic molecules on both external and internal surfaces. As a result, HNTs@Alg-1 and K@Alg-1 beads were selected for further experiments.

As shown in Figure 12b, increasing the bead dosage from 4 mg to 40 mg resulted in a significant decrease in loading capacity, from 58.43 ± 1.7 to 8.18 ± 0.4 mg/g for HNTs@Alg-1 and from 68.74 ± 2.1 to 8.98 ± 0.3 mg/g for K@Alg-1. This decline can be attributed to the limited availability of CTC molecules in the solution, leading to a reduced adsorption mass transfer rate as the bead dosage increased. Based on these findings, 4 mg of HNTs@Alg-1 beads was determined to be the optimal dosage for this study [32].

As depicted in Figure 12c, the highest CTC adsorption on HNTs@Alg and K@Alg beads occurred at pH 3–6, followed by a significant decline as pH increased. Maximum adsorption capacities were observed at approximately pH 4–6 for HNTs@Alg (59.85 ± 2.3 mg/g), and K@Alg (68.74 ± 2.1 mg/g), which aligns with trends reported in previous studies [33].

This behavior can be attributed to the amphoteric nature of CTC, which contains multiple ionizable functional groups. In aqueous solutions, CTC undergoes protonation-deprotonation reactions depending on the solution pH, existing as CTCH_3_^+^, CTCH_2_^±^, CTCH^−^, and CTC^2−^ at pH < 3.30, 3.30 < pH < 7.44, 7.44 < pH < 9.27, and pH > 9.27, respectively [34].

**Figure 13:**
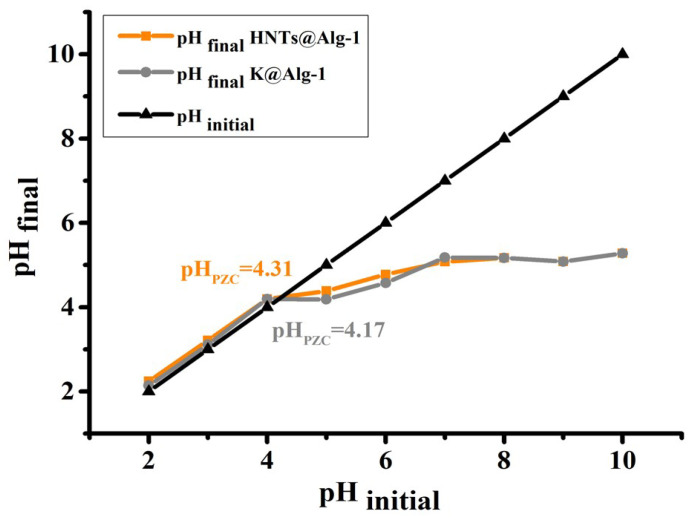
Point of zero charge (pH_PZC_) of HNTs@Alg-1 and K@Alg-1 beads.

The pH_PZC_ of the beads was determined to be approximately 4 (Figure 13). When the solution pH is below the pH_PZC_, the adsorbent surface carries a positive charge, whereas at pH values above the pH_PZC_, it becomes negatively charged [35]. At low pH (pH < pH_PZC_), strong electrostatic repulsion between the protonated CTC species (CTCH^3+^) and the positively charged bead surface inhibited CTC adsorption, resulting in low loading efficiency. Conversely, in the pH range of 4–6, CTC molecules predominantly exist in their neutral or zwitterionic form (CTCH^2±^), which reduces electrostatic repulsion and facilitates interaction with negatively charged adsorption sites through electrostatic attraction (–X^−^–CTCH^2±^). Consequently, the enhanced interaction between CTC and the adsorbent surface led to higher adsorption capacities.

Despite the expected electrostatic repulsion under strongly acidic (pH < 4) or basic (pH 6–10) conditions, the loading capacity remained relatively high. This observation suggests that, in addition to electrostatic attraction between CTCH^2±^ species and the negatively charged surface, hydrogen bonding, complexation, and the physical entrapment of CTC molecules within the lumen of the HNTs also contributed significantly to the adsorption process [36]. At alkaline pH (>10), however, the adsorption capacity sharply decreased to 5.93 ± 0.2 mg/g for HNTs@Alg and 2.98 ± 0.1 mg/g for K@Alg, due to strong electrostatic repulsion between the negatively charged CTC^2−^ species and the bead surfaces [13,37].

In this study, the effect of temperature (295–328K) on CTC adsorption was evaluated (Figure 12d). The results showed that the loading capacity slightly decreases from 58.43 ± 1.7 to 38.08 ± 1.1 mg/g for HNTs@Alg-1 and from 68.74 ± 2.1 to 36.18 ± 0.7 mg/g for K@Alg-1, as temperature increases from 295 to 328 K, indicating an exothermic adsorption process. This suggests that interactions between the clays@alginate beads and CTC are more favorable at lower temperatures, leading to the selection of 295 K as the optimal temperature for subsequent experiments [38].

The influence of contact time on adsorption was also investigated to understand the adsorption kinetics of CTC onto the prepared composites (Figure 12e). The adsorption process was found to be time-dependent, with two distinct phases observed for HNTs@Alg-1. In the initial phase, a rapid increase in adsorption capacity was recorded within the first few minutes, which can be attributed to the availability of abundant vacant active sites on the external surfaces of the beads, facilitating fast CTC uptake. This phase was followed by a slower adsorption phase, where the adsorption rate gradually decreased and reached equilibrium at approximately 6 h. This transition is likely due to the progressive saturation of easily accessible binding sites, leading to molecular diffusion-driven adsorption into less accessible regions, such as the lumen of HNTs or deeper into the alginate matrix. The diffusion limitation at this stage contributes to the slower adsorption rate before reaching equilibrium. A similar trend was observed for K@Alg-1, indicating that both composites follow comparable adsorption kinetics, where external surface interactions dominate the initial phase, and intra-particle diffusion governs the later stage [39,40].

**Figure 14:**
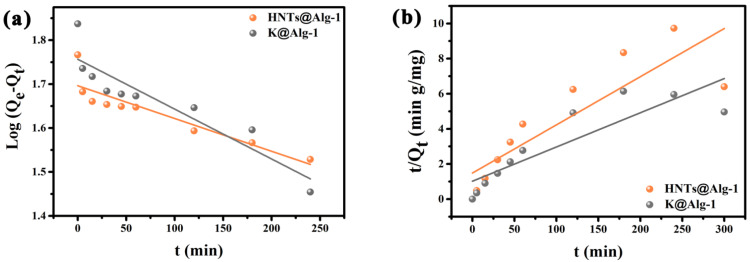
Linear plots of adsorption kinetics for CTC loading on HNTs@Alg-1 and K@Alg-1: (**a**) pseudo-first-order model; (**b**) pseudo-second-order model.

To investigate the adsorption kinetics and elucidate the underlying adsorption mechanism, two widely used linear kinetic models, the pseudo-first-order (PFO) and pseudo-second-order (PSO) models, were employed (Figure 14). These models are mathematically expressed as follows [41]:

(1) $$\log(Q_{e\;-\;}Q_{t})=logQ_{e}-\;k_{1}\frac{t}{2.303}$$

(2) $$\frac{t}{Q_{t}}=\frac{1}{\left(k_{2}Q_{e^{2}}\right)}+\frac{t}{Q_{e}}$$

where *Q_e_* and *Q_t_* (mg/g) represent the adsorption capacities at equilibrium and at time *t*, respectively. *k*_1_ (min^−1^) and *k*_2_ (g/mg·min) denote the rate constants of the pseudo-first-order and pseudo-second-order models, respectively.

As summarized in Table 1, the pseudo-first-order model demonstrates a higher correlation coefficient (*R*^2^) than the pseudo-second-order model. Furthermore, the theoretical equilibrium adsorption capacity predicted by the pseudo-first-order model closely matches the experimentally determined value. These results indicate that the adsorption kinetics of HNTs@Alg-1 and K@Alg-1 for CTC loading conform to the pseudo-first-order model, suggesting that the process is predominantly governed by physisorption mechanisms [13].

Furthermore, the effect of initial CTC concentration (20–100 mg/L) on adsorption performance was examined (Figure 12f). The results revealed that adsorption capacity increased sharply as the initial CTC concentration increased from 20 to 80 mg/L. This increase is due to the higher concentration gradient, which enhances mass transfer and adsorption driving forces, leading to more CTC molecules being captured by the available adsorption sites. However, when the CTC concentration exceeded 80 mg/L, the adsorption curve plateaued, indicating that the adsorption sites had reached saturation, and no further significant increase in adsorption capacity was observed. This behavior suggests a monolayer adsorption mechanism, where once all active binding sites are occupied, additional CTC molecules remain unadsorbed in the solution. The adsorption isotherms align with established models in the literature, further validating the observed adsorption behavior [42].

Table 2 shows that K@Alg-1 and HNTs@Alg-1 beads exhibit a significantly higher loading capacity compared to the unmodified clays and pure Alg beads. This indicates that the incorporation of clays markedly enhances the CTC loading capacity by providing additional binding sites within the alginate matrix.

### 2.3. In Vitro Release Studies

**Figure 15:**
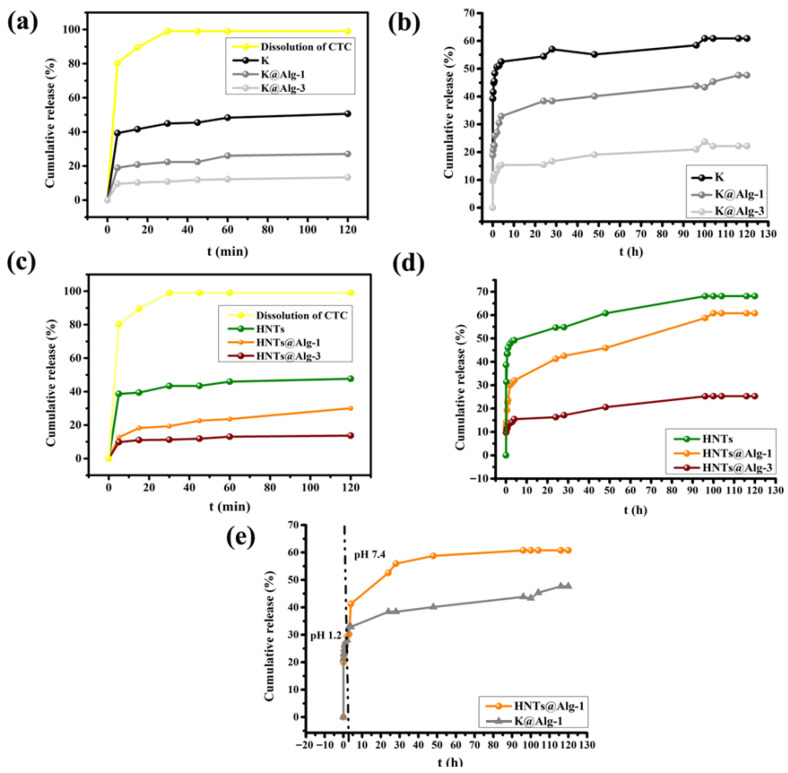
Release profiles of CTC from the studied materials and dissolution profile of free CTC in pH 7.4: (**a**,**c**) Over the first 2 h; (**b**,**d**) Up to 5 days; and (**e**) Release profile of CTC from HNTs@Alg-1 and K@Alg-1 in pH 1.2 followed by pH 7.4.

The in vitro release profiles of CTC from HNTs, HNTs@Alg-1, HNTs@Alg-3, and free CTC were evaluated in phosphate-buffered saline (PBS, pH 7.4) at 37 °C (Figure 15c,d). Free CTC exhibited a rapid release, reaching nearly complete dissolution within 2 h, whereas the nanocomposite formulations displayed sustained release profiles. The cumulative drug release from HNTs, HNTs@Alg-1, and HNTs@Alg-3 after 2 h was approximately 47%, 30%, and 13%, respectively, following the release order: HNTs > HNTs@Alg-1 > HNTs@Alg-3. A similar trend was observed for K and its alginate-based composites (Figure 15a,b), with cumulative release reaching 50%, 27%, and 13% for K, K@Alg-1, and K@Alg-3, respectively. Extended release studies over 120 h revealed significant differences among all samples. Drug liberation continued up to 5 days, with HNTs and K achieving 54% release within 24 h, attributed to their hydrophilic nature and the electrostatic repulsion between negatively charged CTC and the clay surfaces at pH 7.4 [38,43].

The alginate content played a crucial role in modulating release kinetics, where higher alginate concentrations resulted in slower drug diffusion due to enhanced hydrogen bonding and reduced water permeability. Conversely, decreasing the alginate content from 2% to 1% facilitated greater drug release due to reduced intraparticle diffusion resistance and increased water sorption capacity [44]. HNTs@Alg-1 exhibited sustained release, achieving 60.76% compared to only 25.27% for HNTs@Alg-3, indicating that optimizing the Alg concentration is crucial to modulate the release of CTC. The cumulative drug release from K@Alg-1 was 47.66% which means HNTs@Alg-1 perform well in the release of CTC. One probable factor responsible for the observed differences is the tubular structure of halloysite that allow a more sustained diffusion of CTC.

To evaluate the feasibility of using these carriers for potential colon-targeted drug delivery, in vitro studies were conducted to investigate the release kinetics of CTC from HNTs@Alg-1 and K@Alg-1 beads under conditions simulating the human oral route. The CTC-loaded beads were initially placed in a simulated gastric medium (pH 1.2) for 2 h, followed by transfer to a simulated intestinal medium (pH 7.4) for 120 h. The in vitro drug release studies showed that approximately 30% of CTC was released from both composite beads within the first 2 h in the gastric medium. After this period, the HNTs@Alg-1 and K@Alg-1 beads began to disintegrate, releasing the remaining drug in the intestinal environment over the following 120 h, with cumulative release values of approximately 60% and 47%, respectively (Figure 15e). These results suggest that the prepared carriers are not suitable for colon-targeted delivery due to the significant release of the drug in the gastric environment, which compromises their efficiency for this purpose.

Nevertheless, CTC loaded into HNTs@Alg or K@Alg clearly slows the release rate, indicating that these matrices could be useful for the controlled release of drugs aimed at reducing infection and inflammation. Therefore, they may also be considered promising candidates for use as wound dressing materials for long-term treatment applications.

### 2.4. Kinetic Modeling of Drug Release

**Figure 16:**
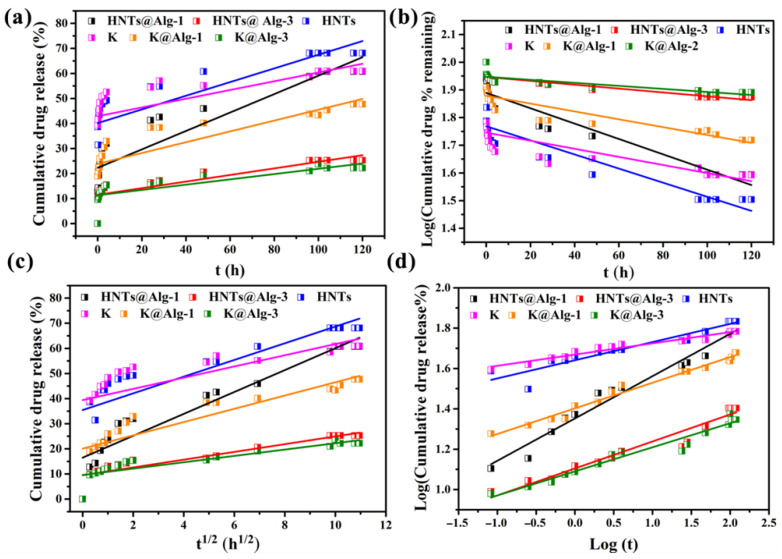
In vitro release profiles of CTC from different formulations: HNTs, HNTs@Alg-1, HNTs@Alg-3, K, K@Alg-1, and K@Alg-3, fitted to various kinetic models. (**a**) Zero-order kinetic model; (**b**) First-order kinetic model; (**c**) Higuchi kinetic model; (**d**) Korsmeyer–Peppas kinetic model.

To better understand the drug release mechanism, four kinetic models, including zero-order, first-order, Higuchi, and Korsmeyer–Peppas, were applied. Figure 16 presents the drug release profiles at pH 7.4 fitted to these models, and the corresponding kinetic parameters are summarized in Table 3. Based on the correlation coefficient (*R*^2^) values, the release of CTC is not well described by the zero-order or first-order kinetic models. In contrast, the Korsmeyer–Peppas model showed the best fit, with the highest *R^2^* values, approaching 0.9. A key parameter in the Korsmeyer–Peppas model is the diffusion exponent (n), which helps identify the drug release mechanism. When n ≤ 0.5, the release is governed by Fickian diffusion. Values of 0.5 < n < 1.0 indicate non-Fickian (anomalous) transport, and n ≥ 1.0 suggests a case II transport mechanism controlled by matrix dissolution and pore collapse [45]. According to Table 3, the calculated n values ranged from 0.090 to 0.210 (n < 0.5), indicating that the release of CTC follows a Fickian diffusion mechanism through a partially swollen matrix and/or water-filled pores. Once the swelling process reaches equilibrium, the system stabilizes, leading to a steady cumulative release of the drug, with no significant concentration gradient between the hydrogel matrix and the release medium [46].

### 2.5. Evaluation of Antibacterial Activity

**Figure 17:**
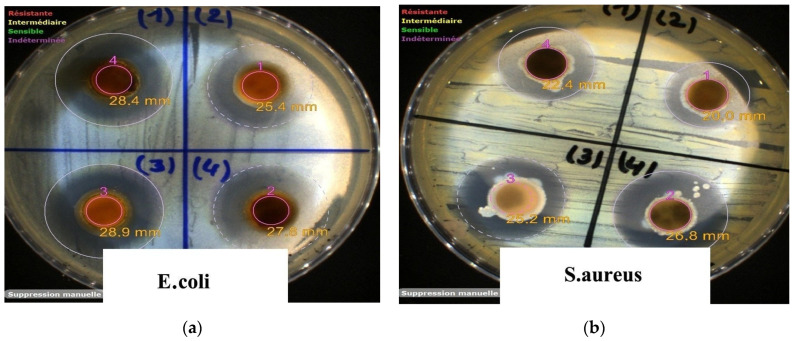
Photographic images showing inhibition zones produced by (1) HNTs@Alg-1–CTC beads, (2) K@Alg-1–CTC beads, (3) Alg–CTC beads, and (4) free CTC against (**a**) *E. coli* and (**b**) *S. aureus*.

**Figure 18:**
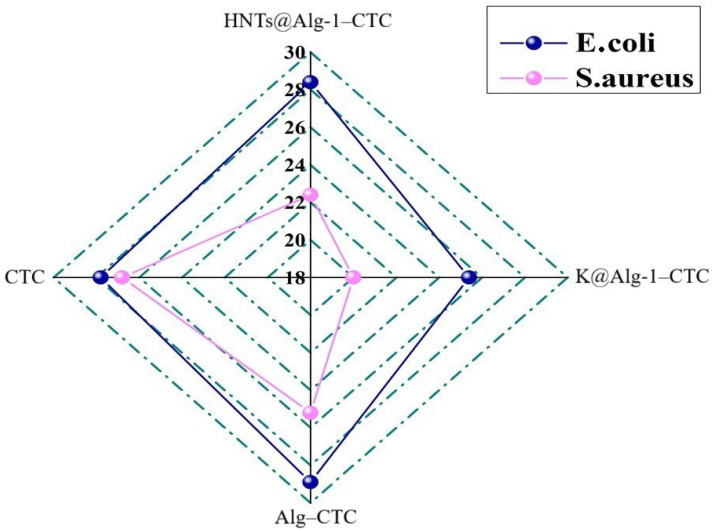
Diameter of inhibition zones illustrating the antibacterial performance of CTC, Alg–CTC, HNTs@Alg-1–CTC, and K@Alg-1–CTC beads against *E. coli* and *S. aureus*.

The agar diffusion assay was used to assess the antibacterial performance of the bead formulations against a Gram-negative strain (*E. coli*) and a Gram-positive strain (*S. aureus*). *E. coli* is commonly implicated in gastrointestinal and urinary tract infections, whereas *S. aureus* is known for producing enterotoxins associated with foodborne illness [47]. As shown in Figure 17, all bead systems effectively delivered CTC, producing distinct inhibition zones (Figure 18). The Alg–CTC beads exhibited the strongest initial antibacterial response, with inhibition zones of 28.9 mm against *E. coli* and 25.2 mm against *S. aureus*. This pronounced activity is consistent with a burst-release effect, in which a substantial portion of the antibiotic is rapidly liberated from the hydrogel matrix.

The HNTs@Alg-1 beads demonstrated similarly high activity (28.4 mm and 22.4 mm), notably outperforming the K@Alg-1 beads (25.4 mm and 20.0 mm). The superior performance of the HNTs-Alg composite correlates with its enhanced drug-release behavior, which resulted in a higher cumulative release of CTC and consequently greater radial diffusion into the agar medium. In contrast, the kaolin-based beads retained the antibiotic more strongly, limiting its diffusion and leading to smaller inhibition zones, an outcome consistent with their release efficiency.

## 3. Conclusions

In this study, alginate-based composite beads incorporating halloysite nanotubes and kaolin were developed and characterized as drug delivery systems using chlortetracycline as a model drug. Structural analyses confirmed the integration of the clays into the alginate matrix without altering their native structures, preserving functional groups essential for drug interaction. Lower alginate concentrations produced more porous beads, enhancing drug loading and diffusion. The optimized formulations, HNTs@Alg-1 and K@Alg-1, showed high drug loading capacities (59.85 ± 2.3 mg/g and 68.74 ± 2.1 mg/g), attributed to favorable surface interactions and porosity. Drug incorporation occurred via electrostatic interactions, hydrogen bonding, and complexation. Adsorption was most effective at mildly acidic pH (4–6) and lower temperatures, following a two-stage kinetic pattern with rapid initial uptake followed by slower diffusion, highlighting the influence of matrix design on drug adsorption efficiency.

In vitro release studies revealed that free CTC dissolved almost completely within 2 h, while composite formulations, particularly HNTs@Alg-1, exhibited significantly sustained release, with 60.76% cumulative release over 120 h, outperforming K@Alg-1 (47.66%) and HNTs@Alg-3 (25.27%). Increasing alginate concentration reduced drug diffusion due to decreased water permeability, and the tubular structure of HNTs contributed to prolonged release. About 30% of CTC was prematurely released in simulated gastric fluid, limiting their suitability for colon-targeted delivery. The sustained release observed in alkaline conditions highlights their potential for long-term therapeutic use in treating infections and inflammation. Kinetic modeling revealed Fickian diffusion as the primary release mechanism. Among the applied models, the Korsmeyer–Peppas model best described the release behavior.

In summary, HNTs@Alg and K@Alg beads represent a promising platform owing to their favorable structural and chemical properties, effective drug adsorption, and sustained-release performance. Antibacterial assays further demonstrated that the CTC-loaded formulations effectively inhibited *E. coli* and *S. aureus*, confirming that the drug retained its bioactivity after encapsulation and release. The combined sustained-release profile and antibacterial properties highlight their potential as low-cost, biocompatible materials for wound healing and infection-control applications. Future work will include cytotoxicity, hemocompatibility, and anti-inflammatory evaluations to further confirm their therapeutic potential.

## 4. Materials and Methods

Natural halloysite nanotubes (HNTs) were obtained as raw clay from the Djebel Debbagh deposit in Guelma, Algeria. The material was supplied by SOALKA (Algerian Company of Kaolins), El Milia, Jijel, Algeria. Kaolin clay was purchased from Biochem Chemopharma, Paris, France. Additional reagents, including sodium alginate (SA) (NaC_6_H_7_O_6_), sodium hydroxide (NaOH), hydrochloric acid (HCl), Sodium Chloride (NaCl), and calcium chloride (CaCl_2_), were procured from Sigma-Aldrich, Shanghai, China. Chlortetracycline (C_22_H_24_C_l2_N_2_O_8_·HCl), with a molecular weight of 515.3 g/mol, was obtained from Merck, Darmstadt, Germany.

### 4.1. Preparation of Alginate Composite Beads Based on K and HNTs

**Figure 19:**
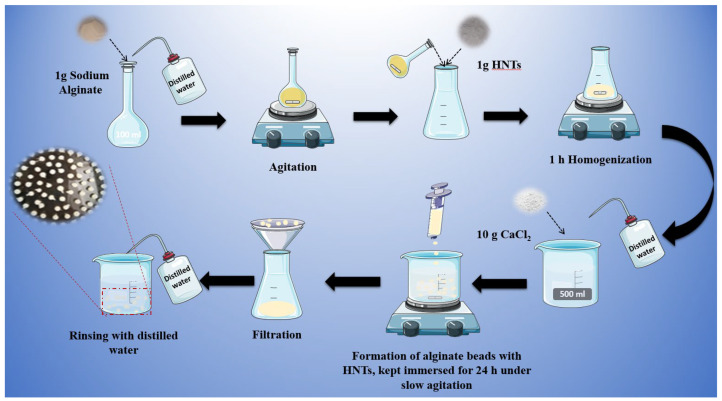
Preparation method of HNTs@Alg-1.

**Figure 20:**
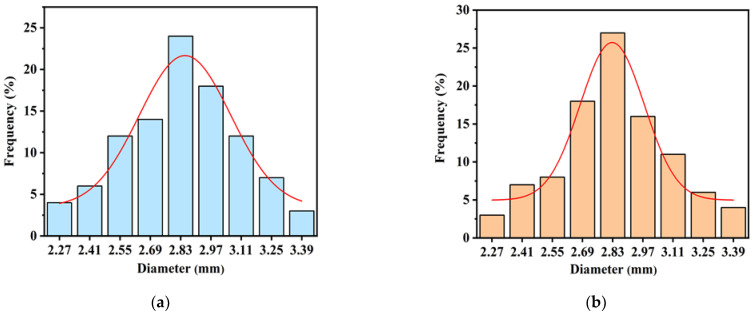
Particle size distribution of (**a**) K@Alg-1 beads and (**b**) HNTs@Alg-1 beads.

Alginate-based composite beads with different compositions, incorporating K and HNTs, were synthesized via a simple solution-mixing method in the presence of Ca^2+^ ions [22]. Taking HNTs@Alg-1 as a representative formulation, the synthesis procedure (Figure 19) was as follows: A 1% (*w*/*v*) SA solution was prepared by dissolving 1 g of in 100 mL of double-distilled (DD) water under continuous stirring. Subsequently, 1 g of HNTs was added to the alginate solution and stirred vigorously at room temperature for 1 h to ensure uniform dispersion. The resulting suspension was then carefully dripped into 500 mL of an aqueous CaCl_2_ solution (2%, *w*/*v*) to facilitate gelation. The formed beads were allowed to age undisturbed in the CaCl_2_ solution for up to 24 h to enhance their structural integrity. Finally, the HNTs-loaded alginate beads were dried in a hot air oven at 60 °C for 12 h. After solidification, the beads were thoroughly rinsed with distilled water to remove excess CaCl_2_, yielding raw beads with an average diameter of approximately 2.8 mm (Figure 20). For comparison, blank alginate beads (1% SA) were prepared following the same procedure but without the addition of any clay (K or HNTs). The designation, composition, and yield of the synthesized formulations are summarized in Table 4. The percentage yield was calculated as the ratio of the total weight of the dried composite beads obtained from each batch to the total weight of the raw materials used in their preparation, namely sodium alginate and clay (halloysite or kaolin) [48]. The yield was determined using the following equation:

(3) $$Percentage\;Yield\;\left(\%\right)=\frac{Weight\;of\;dried\;beads}{Weight\;of\;sodium\;alginate\;+\;Weight\;of\;clay}\times 100$$

The process yield of the beads ranged from 85 ± 1.25% to 89 ± 2.15%, indicating a highly efficient and reproducible bead formation process.

### 4.2. Characterization Studies

Scanning electron microscopy (SEM) analysis was performed at ATOMKI using a dual-beam scanning electron microscope (focused ion beam–scanning electron microscopy (FIB-SEM), Thermo Fisher Scientific, Waltham, MA, USA, Model: Scios 2). The system was equipped with a Bruker Quantax Energy Dispersive X-ray (EDS) detector for compositional analysis, enabling the examination of the surface morphology of both the raw clays and the synthesized beads. Elemental composition analysis was performed using EDS integrated with the SEM. The samples were analyzed both before and after their interaction with CTC. Fourier transform infrared (FT-IR) spectroscopy was performed using an FT-IR-8400 spectrometer (SHIMADZU) to investigate the structural characteristics of K, HNTs, and the synthesized beads, both before (fresh) and after (spent) CTC adsorption. Spectra were recorded using the potassium bromide (KBr) pellet technique at a resolution of 2 cm^−1^ over a frequency range of 4000–400 cm^−1^. The phase structures of the raw clay and fresh beads were determined using X-ray diffraction (XRD) with a Bruker D8 ADVANCE/Ultima IV diffractometer equipped with Cu Kα radiation (λ = 0.15405 nm). The analysis was conducted at an operating voltage of 40 kV and a current of 30 mA, scanning over a 2θ range of 5–90° at a speed of 8°/min. X-ray photoelectron spectroscopy (XPS) was performed to investigate the surface chemical composition of the samples. Measurements were carried out using an XR 50 dual-anode, non-monochromatized X-ray source and a Phoibos 100 MCD-5 hemispherical energy analyzer (SPECS, Berlin, Germany). Prior to analysis, the samples were oven-dried at 120 °C, ground in a mortar, and dusted onto double-sided adhesive tape affixed to copper sample holders. They were then introduced into the high vacuum loading chamber (10^–7^ mbar) and left to degas overnight before being transferred to the spectrometer. The base vacuum in the instrument was 5 × 10^–10^ mbar, and approximately 1 × 10^–9^ mbar during measurements. Samples were indirectly cooled by liquid nitrogen before and throughout X-ray exposure. Spectra were sequentially recorded using Al Kα radiation (1486.6 eV) at 10 kV acceleration voltage and 10 mA emission current (100 W X-ray power). The binding energy scale was calibrated using the Au 4f and Cu 2p peaks of a freshly cleaned reference sample containing both metals, as prescribed by the manufacturer. Spectral analysis was performed using CasaXPS software (v2.3.25PR1.0). Charge referencing was performed using the C 1s peak of carbon at 284.8 eV. While this method has known limitations, as discussed by Greczynski and Hultman [49], alternative referencing approaches were not feasible due to the nature of the samples.

### 4.3. Determination of the pH_PZC_ of HNTs@Alg-1 and K@Alg-1

The pH_PZC_ of the prepared beads was evaluated by employing the salt addition method. In brief, 0.5 g of each bead sample was introduced into 20 mL of a 0.01 M NaCl solution, whose pH was pre-adjusted using HCl or NaOH. The suspensions were maintained under continuous agitation at 160 revolutions per minute (rpm) and 298 K for 24 h to ensure equilibrium. After centrifugation, the equilibrium pH of each solution was recorded. The pH_PZC_ of HNTs@Alg-1 and K@Alg-1 was determined from the intersection point of the plot of final pH versus initial pH [50].

### 4.4. CTC Loading onto Alginate Composite Beads Based on K and HNTs

To load CTC onto raw K, HNTs, and alginate-based composite beads, synthesized HNTs@Alg-n (n = 1, 2, 3, 4) and K@Alg-n (n = 1, 2, 3, 4) were immersed in 10 mL of a 100 mg/L CTC solution at pH 4 and 22 °C for 4 h under continuous shaking at 200 rpm. Following the adsorption process, the residual CTC concentration in the solution was determined using a UV-Vis spectrophotometer. The drug loading capacity per dry weight of the beads was calculated using Equation (4):

(4) $$Q=\frac{\left(Co-Ce\right)\times V}{m}$$

where *Q* (mg CTC/g dry beads) represents the drug loading (adsorption) capacity, *C_0_* (mg/L) is the initial CTC concentration, *C_e_* (mg/L) is the equilibrium CTC concentration, *V* (L) is the volume of the CTC solution, and *m* (g) is the dry mass of the adsorbent.

To determine the optimal drug loading capacity, various parameters were systematically evaluated, including adsorbent dosage (4–40 mg), solution pH (2–12), temperature (295–328 K), contact time (5 min to 6 h), and initial CTC concentration (10–100 mg/L) [51,52]. Each experiment was conducted in triplicate to ensure reproducibility, and the results were averaged.

### 4.5. In Vitro Drug Release Study

The drug release behavior of the loaded samples was investigated in buffer media at different pH levels (pH 7.4 and 1.20), following the method previously reported by Kumar et al. For the release experiments, a known quantity of drug-loaded HNTs, K, and HNTs/K-alginate composite beads was immersed in 100 mL of the respective release medium (pH 1.20 or 7.40) and stirred at 100 rpm. At predetermined time intervals (ranging from 5 min to 120 h), 4 mL aliquots were withdrawn from each solution and replaced with an equal volume of fresh buffer to maintain a constant volume. The collected samples were analyzed using an ultraviolet–visible (UV–Vis) spectroscopy to determine the drug concentration and evaluate the release profile, as calculated by Equation (5) [53].

(5) $$\%\;Release=\frac{Released\;CTC}{Total\;CTC}\times 100$$

### 4.6. Drug Release Kinetics

The kinetics of CTC release from the synthesized beads were evaluated by fitting the release profiles to four different mathematical models [38,53]:

- Zero-order model:

(6) $$M_{t}=M_{0}+K_{0}t$$

- First order model:

(7) $$logM_{t}=logM_{0}-K_{1}t/2.303$$

- Higuchi model:

(8) $$M_{t}/M_{\infty }=K_{H}t^{1/2}$$

- Korsmeyer–Peppas model:

(9) $$\log(M_{t}/M_{\infty })=\log K_{kp}+n\log t$$

where *M_t_* is the cumulative amount of drug released at time *t*, *M_∞_* is the maximum drug release, *K* is the release rate constant for each model, and *n* is a release exponent that indicates the mechanism of drug diffusion.

### 4.7. Antibacterial Activity

The antibacterial potential of CTC, Alg–CTC, HNTs@Alg-1–CTC, and K@Alg-1–CTC beads was evaluated using the agar diffusion method. The tests were performed against Escherichia coli (Gram-negative, American Type Culture Collection (ATCC) 25923) and Staphylococcus aureus (Gram-positive, ATCC 25922). In this procedure, bacterial suspensions adjusted to a 0.5 McFarland standard were uniformly spread over the surface of solid agar plates. The prepared beads were then aseptically placed onto the inoculated agar surfaces, and the plates were incubated at 37 °C for 24 h. After incubation, antibacterial activity was assessed by measuring the diameter of the inhibition zones surrounding each sample (in millimeters). The observed inhibition zones were used to evaluate the antibacterial performance of the tested materials [54].

## Figures and Tables

**Table 1:** Adsorption kinetic parameters of HNTs@Alg-1 and K@Alg-1 for CTC loading.

Models	Parameters	Adsorbents	
		HNTs@Alg-1	K@Alg-1
PFO	*k*_1_ (min^−1^)	0.0017	0.0026
	Q*_e_*_,*exp*_ (mg/g)	58.4367	68.7409
	Q*_e_*_,*cal*_ (mg/g)	49.700	57.1018
	*R* ^2^	0.8050	0.8269
PSO	*k*_2_ (g/mg min)	0.0005	0.0003
	Q*_e_*_,*exp*_ (mg/g)	58.4369	68.7409
	Q*_e_*_,*cal*_ (mg/g)	36.4564	51.3347
	*R* ^2^	0.7226	0.7528

**Table 2:** Loading capacity of CTC on Alg, K, HNTs, K@Alg-1, and HNTs@Alg-1 beads.

	Alg	K	HNTs	K@Alg-1	HNTs@Alg-1
Q (mg/g)	52.08 ± 2.4	40.1 ± 1.2	48.12 ± 1.4	68.74 ± 2.1	59.85 ± 2.3

**Table 3:** Kinetic parameters and correlation coefficients (R^2^) obtained from modeling the release of CTC using zero-order, first-order, Higuchi, and Korsmeyer–Peppas models.

Materials	Zero-Order		First-Order		Higuchi		Korsmeyer Peppas		
	K_0_ (h^−1^)	R_1_^2^	K_1_ (h^−1^)	R_2_^2^	K_H_ (h^−0.5^)	R_3_^2^	K_KP_ (h^−n^)	n	R_4_^2^
HNTs	0.273 ± 0.062	0.535	0.006 ± 8.6 × 10^−4^	0.738	7.556 ± 1.047	0.750	43.742 ± 1.023	0.090 ± 0.007	0.907
HNTs@Alg-1	0.368 ± 0.045	0.803	0.006 ± 5.5 × 10^−4^	0.894	6.326 ± 0.504	0.902	22.504 ± 1.026	0.210 ± 0.008	0.978
HNTs@Alg-3	0.132 ± 0.018	0.765	0.002 ± 2 × 10^−4^	0.805	2.680 ± 0.281	0.841	12.719 ± 1.025	0.134 ± 0.008	0.949
K	0.174 ± 0.064	0.288	0.003 ± 9.1 × 10^−4^	0.444	6.938 ± 1.161	0.671	46.669 ± 1.010	0.056 ± 0.003	0.956
K@Alg-1	0.216 ± 0.041	0.626	0.003 ± 5.1 × 10^−4^	0.717	5.040 ± 0.599	0.804	25.256 ± 1.014	0.127 ± 0.004	0.982
K@Alg-3	0.106 ± 0.018	0.670	0.001 ± 2 × 10^−4^	0.710	2.410 ± 0.285	0.806	12.304 ± 1.021	0.119 ± 0.007	0.957

**Table 4:** Designation and composition of synthesized alginate-based composite beads and their process yield.

Designation	Description	Concentration of SA (%)	Mass of K or HNTs (g)	Process Yield
HNTs@Alg-1	Halloysite nanotube-alginate beads	1	1	85 ± 1.25
HNTs@Alg-2	Halloysite nanotube-alginate beads	1	2	86 ± 2.45
HNTs@Alg-3	Halloysite nanotube-alginate beads	2	2	89 ± 1.16
HNTs@Alg-4	Halloysite nanotube-alginate beads	2	1	86 ± 1.25
K@Alg-1	Kaolin-alginate beads	1	1	85 ± 2.32
K@Alg-2	Kaolin-alginate beads	1	2	89 ± 2.15
K@Alg-3	Kaolin-alginate beads	2	2	86 ± 2.51
K@Alg-4	Kaolin-alginate beads	2	1	85 ± 3.25
Alg	Alginate beads	1	-	87 ± 1.75

## Data Availability

The data presented in this study are available in the article and Supplementary Material.

## Supplementary Materials

The following supporting information can be downloaded at: https://www.mdpi.com/article/10.3390/gels11110921/s1, Figure S1: FT-IR Spectra of free CTC drug showing characteristic functional groups.

## Abbreviations

The following abbreviations are used in this manuscript:

Alg	Alginate
HNTs	Halloysite Nanotubes
K	Kaolin
Ca^2+^	Calcium Ions
HNTs@Alg	Halloysite Nanotubes–Alginate Composite Beads
K@Alg	Kaolin–Alginate Composite Beads
FTIR	Fourier Transform Infrared Spectroscopy
SEM-EDS	Scanning Electron Microscopy with Energy-Dispersive Spectroscopy
XRD	X-ray Diffraction
XPS	X-ray Photoelectron Spectroscopy
CTC	Chlortetracycline Hydrochloride
DDS	Drug Delivery System(s)
TC	Tetracycline Hydrochloride
MWCNTs	Multiwall Carbon Nanotubes
GO	Graphene Oxide
LBL	Layer-by-Layer
Si–O–Si	Siloxane Group
Al–OH	Aluminum Hydroxyl Group
Ca	Calcium
O	Oxygen
C	Carbon
Al	Aluminum
Si	Silicon
CaCl_2_	Calcium Chloride
NaOH	Sodium Hydroxide
HCl	Hydrochloric Acid
SA	Sodium Alginate
DD	Double-Distilled (Water)
UV–Vis	Ultraviolet–Visible Spectroscopy
PBS	Phosphate-Buffered Saline
FIB-SEM	Focused Ion Beam–Scanning Electron Microscope
MIL-100(Fe)	Metal–Organic Framework MIL-100 Containing Iron
°C	Degrees Celsius
rpm	Revolutions Per Minute
K (in temperature)	Kelvin
eV	Electron Volt
R^2^	Coefficient of Determination
n	Diffusion Exponent (in Korsmeyer–Peppas Model)
KBr	Potassium Bromide
N	Nitrogen
Cu	Copper
NaCl	Sodium Chloride
pH_PZC_	Point of zero charge
m	Mass
Q	Capacity
C_0_	Initial concentration
Cₑ	Equilibrium concentration
V	Volume
PFO	Pseudo-First-Order Model
PSO	Pseudo-Second-Order Model
*E. coli*	*Escherichia coli*
*S. aureus*	*Staphylococcus aureus*
ATCC	American Type Culture Collection
