# Supplementary material for: Alginate–Aluminosilicate Clay Beads for Sustained Release of Chlortetracycline Hydrochloride: Development and In Vitro Studies
Source: Gels. 2025 Nov 18;11(11):921. doi: 10.3390/gels11110921 (PMC12652813; doi:10.3390/gels11110921)
Supplement: Supplementary file 1 [file gels-11-00921-s001.zip › gels-3971958-supplementary.pdf]

Alginate–Aluminosilicate Clay Beads for Sustained Release of Chlortetracycline Hydrochloride: Development and In-Vitro Studies

Aicha Nour Laouameria [1,2,3,*,‡], Meriem Fizir [3,4,*,‡], Sami Touil [3], Amina Richa [3], Nassima Benamara [3], Houda Douba [4,5], Liu Wei [6], Djamila Aouameur [3,4], Houria Rezala [4], Attila Csík [2] and Tamás Fodor [2]

[1] Doctoral School of Chemistry, University of Debrecen, Egyetem tér 1, H-4032 Debrecen, Hungary
[2] HUN-REN Institute for Nuclear Research, Bem tér 18/c, 4026 Debrecen, Hungary; csik.attila@atomki.hu; fodor.tamas@atomki.hu
[3] Laboratory of Precision Agriculture, Environment and Sustainable Development, Khemis Miliana University, Algeria; s.touil@univ-db.dz; a.richa@univ-dbkm.dz; nassimabenamara801@gmail.com; d.aouameur@univ-dbkm.dz
[4] Laboratoire de Valorisation des Substances Naturelles, Khemis Miliana University, Algeria; h.douba@univ-dbkm.dz; h.rezala@univ-dbkm.dz
[5] Laboratory of Physical Chemistry of Material Interfaces Applied to the Environment, University of Saad Dahlab Blida 1, Algeria
[6] Zhejiang Pharmaceutical University, Ningbo315500, Zhejiang, China; liuw@zjpc.net.cn
* Correspondence: A.N.L. (laouameria.aicha.nour@atomki.hu); M.F. (meriem.fizir@univ-dbkm.dz)
‡ A.N.L. and M.F. contributed equally to this work

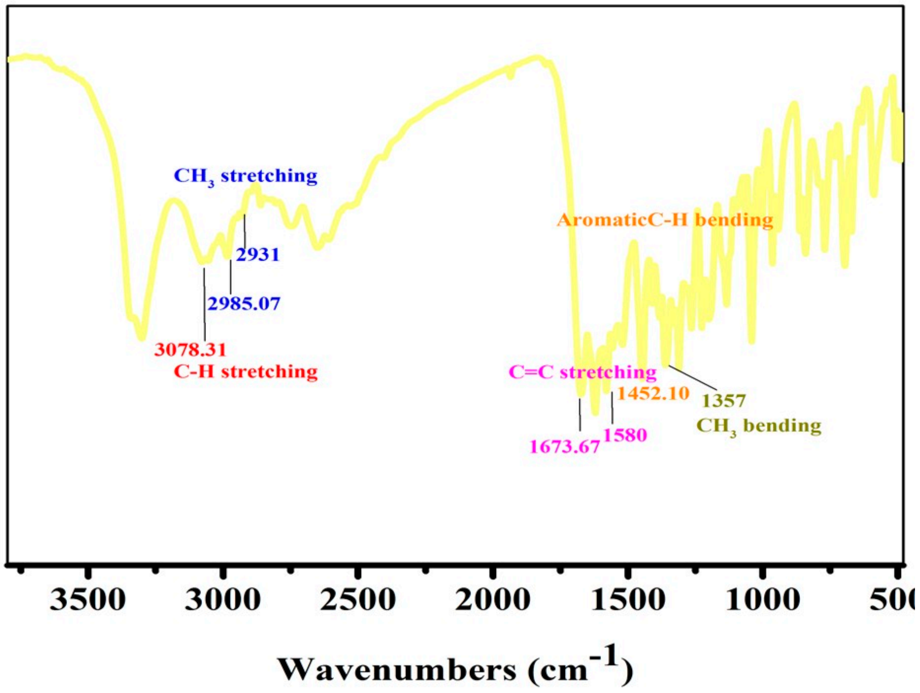

Figure S1. FT-IR Spectra of free CTC drug showing characteristic functional groups.
